# Breaking sitting with light activities vs structured exercise: a randomised crossover study demonstrating benefits for glycaemic control and insulin sensitivity in type 2 diabetes

**DOI:** 10.1007/s00125-016-4161-7

**Published:** 2016-11-30

**Authors:** Bernard M. F. M. Duvivier, Nicolaas C. Schaper, Matthijs K. C. Hesselink, Linh van Kan, Nathalie Stienen, Bjorn Winkens, Annemarie Koster, Hans H. C. M. Savelberg

**Affiliations:** 1 Department of Human Biology and Movement Science, NUTRIM School for Nutrition and Translational Research in Metabolism, Maastricht University Medical Centre+, PO Box 616, 6200 MD Maastricht, the Netherlands; 2 Division of Endocrinology, Department of Internal Medicine, CARIM School for Cardiovascular Diseases, Maastricht University Medical Centre+, Maastricht, the Netherlands; 3 CAPHRI School for Public Health and Primary Care, Maastricht University Medical Centre+, Maastricht, the Netherlands; 4 Department of Methodology and Statistics, Maastricht University Medical Centre+, Maastricht, the Netherlands; 5 Department of Social Medicine, Maastricht University Medical Centre+, Maastricht, the Netherlands

**Keywords:** Glycaemic control, Insulin sensitivity, Light-intensity physical activity, Lipid metabolism, Sedentary behaviour, Sedentary breaks, Standing, Type 2 diabetes, Walking

## Abstract

**Aims/hypothesis:**

We aimed to examine the effects of breaking sitting with standing and light-intensity walking vs an energy-matched bout of structured exercise on 24 h glucose levels and insulin resistance in patients with type 2 diabetes.

**Methods:**

In a randomised crossover study, 19 patients with type 2 diabetes (13 men/6 women, 63 ± 9 years old) who were not using insulin each followed three regimens under free-living conditions, each lasting 4 days: (1) Sitting: 4415 steps/day with 14 h sitting/day; (2) Exercise: 4823 steps/day with 1.1 h/day of sitting replaced by moderate- to vigorous-intensity cycling (at an intensity of 5.9 metabolic equivalents [METs]); and (3) Sit Less: 17,502 steps/day with 4.7 h/day of sitting replaced by standing and light-intensity walking (an additional 2.5 h and 2.2 h, respectively, compared with the hours spent doing these activities in the Sitting regimen). Blocked randomisation was performed using a block size of six regimen orders using sealed, non-translucent envelopes. Individuals who assessed the outcomes were blinded to group assignment. Meals were standardised during each intervention. Physical activity and glucose levels were assessed for 24 h/day by accelerometry (activPAL) and a glucose monitor (iPro2), respectively. The incremental AUC (iAUC) for 24 h glucose (primary outcome) and insulin resistance (HOMA2-IR) were assessed on days 4 and 5, respectively.

**Results:**

The iAUC for 24 h glucose (mean ± SEM) was significantly lower during the Sit Less intervention than in Sitting (1263 ± 189 min × mmol/l vs 1974 ± 324 min × mmol/l; *p* = 0.002), and was similar between Sit Less and Exercise (Exercise: 1383 ± 194 min × mmol/l; *p* = 0.499). Exercise failed to improve HOMA2-IR compared with Sitting (2.06 ± 0.28 vs 2.16 ± 0.26; *p* = 0.177). In contrast, Sit Less (1.89 ± 0.26) significantly reduced HOMA2-IR compared with Exercise (*p* = 0.015) as well as Sitting (*p* = 0.001).

**Conclusions/interpretation:**

Breaking sitting with standing and light-intensity walking effectively improved 24 h glucose levels and improved insulin sensitivity in individuals with type 2 diabetes to a greater extent than structured exercise. Thus, our results suggest that breaking sitting with standing and light-intensity walking may be an alternative to structured exercise to promote glycaemic control in patients type 2 diabetes.

***Trial registration*:**

Clinicaltrials.gov NCT02371239

***Funding*:**

The study was supported by a Kootstra grant from Maastricht University Medical Centre^+^, and the Dutch Heart Foundation. Financial support was also provided by Novo Nordisk BV, and Medtronic and Roche made the equipment available for continuous glucose monitoring

**Electronic supplementary material:**

The online version of this article (doi:10.1007/s00125-016-4161-7) contains peer-reviewed but unedited supplementary material, which is available to authorised users.

## Introduction

Moderate- to vigorous-intensity exercise is one of the cornerstones of prevention and treatment of type 2 diabetes [1, 2], with current physical activity guidelines recommending performance of at least 150 min/week exercise at these intensities [3]. Although this approach has proved effective in the prevention [4] and treatment [5] of type 2 diabetes, more than 90% of healthy adults do not adhere to these guidelines [6]. In view of type 2 diabetes-related comorbidities such as muscle weakness and peripheral neuropathy, which can be a barrier to physical activity [7], non-compliance may be even higher in individuals with type 2 diabetes. Alternatives to exercise are therefore needed for the treatment of type 2 diabetes.

The results of population-based studies suggest that the average adult spends more than half of the waking day sedentary, partaking in activities such as watching television and using the computer [8–10]. Recent evidence from observational studies shows an association between sedentary time and an increased risk of type 2 diabetes, independent of the time spent exercising [8, 10]. Experimental studies under laboratory conditions suggest that regular interruption of sitting using small bouts of walking may be effective in lowering glucose and insulin levels in healthy and overweight/obese adults and in individuals with type 2 diabetes [11–15]. We recently showed that replacing sitting time with standing and light-intensity walking in free-living conditions was more efficient at improving insulin action than replacement with one bout of moderate- to vigorous-intensity exercise (cycling) in healthy sedentary participants [16]. Since energy expenditure was comparable by design during these two conditions, these data suggest that sitting has negative effects on insulin sensitivity independent of energy expenditure.

To investigate whether these findings could be replicated in individuals with type 2 diabetes, we investigated whether, under conditions of comparable energy expenditure, breaking up sitting time with standing and light-intensity walking would improve 24 h glucose levels and insulin sensitivity in type 2 diabetes patients as compared with structured exercise. To our knowledge, this is the first study investigating the glycaemic effects of breaking up sitting time in people with type 2 diabetes in free-living conditions.

## Methods

### Participants

Adults with type 2 diabetes (minimum duration of 1 year), aged 40–75 years and with a BMI of 25–35 kg/m^2^, were recruited through online and paper advertisements. Exclusion criteria were more than 2.5 h/week of moderate- to vigorous-intensity exercise based on self-report, diseases that interfered with physical activity participation, alcohol abuse, experimental drug use and use of insulin, corticosteroids, coumarins or immunosuppressants. Participants were instructed to discontinue lipid-lowering drugs and cholesterol-lowering margarines 14 days prior to starting the first regimen. Use of other drugs was continued and maintained at the same dose during the study. Other exclusion criteria were fasting triacylglycerol >10 mmol/l, fasting plasma glucose ≥11 mmol/l or HbA_1c_ >10% (86 mmol/mol). All participants provided written informed consent. The study was conducted at Maastricht University between February and May 2015 in accordance with the principles of the Declaration of Helsinki, and was approved by the Local Ethics Committee of the Maastricht University Medical Centre^+^ (www.clinicaltrials.gov registration no. NCT02371239).

### Study design

The study used a randomised crossover design, and the analysis of the primary and secondary outcomes was performed without knowledge of which activity regimen the participant had been allocated to. Blocked randomisation was performed using a block size of six regimen orders using sealed, non-translucent envelopes.

The number of participants required to detect a clinically relevant difference in 24 h glucose profiles between the activity regimens was calculated based on a similar study [17] with three study arms (sedentary, low-intensity exercise and high-intensity exercise), in which the mean ± SD 24 h glucose was 8.7 ± 2.1 mmol/l during the high-intensity exercise regimen. Using these data, and assuming equal SDs in each of our regimens, a correlation between repeated measurements of 0.5 and a Bonferroni corrected two-sided alpha of 0.017 (=0.05/3), it was calculated that 19 participants were needed to detect a mean difference of 1.7 mmol/l in 24 h glucose between the activity regimens, with a power of 80%, using a paired-samples *t* test.

### Activity regimens

**Fig. 1:**
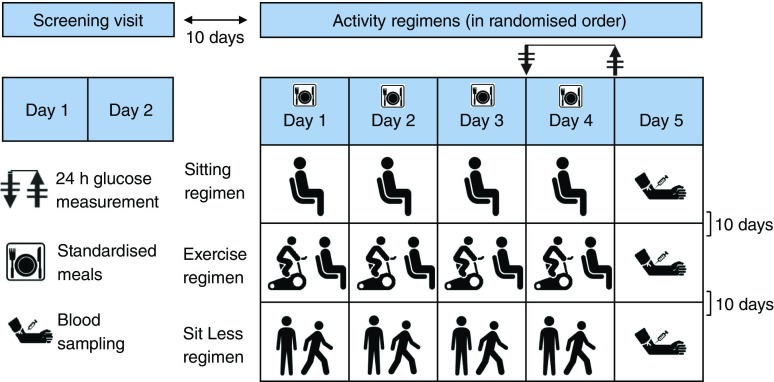
Study design. Each participant followed three activity regimens under free-living conditions, each lasting 4 days: (1) Sitting: 14 h sitting/day; (2) Exercise: 1.1 h/day of sitting replaced by moderate- to vigorous-intensity cycling; and (3) Sit Less: 4.7 h/day of sitting replaced by standing (2.5 h) and light-intensity walking (2.2 h), relative to time spent doing these activities in the Sitting regimen

All participants were instructed to follow three activity regimens (‘Sitting’, ‘Exercise’ and ‘Sit Less’; see Fig. 1). The order of intervention was randomised and each regimen lasted 4 days and was carried out in free-living conditions. During the Sitting regimen, participants were instructed to restrict walking to 1 h/day and standing to 1 h/day while spending the remainder of the waking day (∼14 h) sitting. During the Exercise regimen, about 1 h/day of sitting time was replaced with supervised cycling on an ergometer (Lode Excalibur, Groningen, the Netherlands) at our research centre. To increase the feasibility of the exercise, cycling was performed in the morning at least 2 h after breakfast to prevent (abdominal) discomfort during cycling. Cycling was carried out in 20 min bouts with a 5 min rest (sitting) after each bout. In the Sit Less regimen, participants were instructed to replace approximately 5 h/day sitting with 2 h walking and 3 h standing. Participants were instructed to break up their sitting time, preferably every 30 min, by dividing the walking/standing activities into smaller bouts over the day. They were instructed to walk at a self-perceived light-intensity. Importantly, adherence to these instructions was carefully monitored by advanced accelerometry (see below).

A wash-out period of at least 10 days between the screening session and the first activity regimen, and between successive activity regimens, was applied. During the wash-out phase, participants were instructed to maintain their habitual pattern of daily life activities, not to perform more than 1 h/week of moderate- to vigorous-intensity exercise and to consume a maximum of 1 unit/day of alcohol.

### Meal standardisation

During the first 60 h of each regimen, participants were instructed to adhere to their normal diet. During the last 36 h of each regimen, standardised pre-packaged meals were provided based on the individual’s basal metabolic rate, and matched to each individual’s daily energy requirements (this was calculated by multiplying the basal metabolic rate [determined during screening using a ventilated hood] by 1.4 [reflective of the physical activity level of a sedentary lifestyle]) [18]. During the first regimen, participants carefully recorded everything they ate and drank, as well as the timing of consumptions. Subsequently, these records were returned to the participants, who were instructed to consume the same diet at the same time points during the following two regimens. Alcohol was not permitted during regimen participation.

### Assessment of 24 h glucose, insulin resistance and lipids

During the activity regimens, glucose levels were continuously measured (24 h/day) with a glucose monitor (iPro2 Professional CGM MiniMed; Medtronic, Northridge, CA, USA) connected to a glucose sensor (Enlite Glucose Sensor MiniMed; Medtronic). The sensor was inserted subcutaneously on the first morning of each regimen, at 5 cm from the umbilicus, on the right side of the abdomen. To investigate the cumulative effects of the 4 day activity regimens, 24 h glucose levels were only analysed on day 4; therefore, standardised meals were provided by the research team for dinner on day 3 and for all meals and snacks during day 4.

Glucose concentration was measured in the interstitial fluid of the subcutaneous tissue every 5 min via the iPro2. Additionally, participants collected blood glucose measurements four times per day (before main meals and before sleep) using a capillary glucose meter (Accu-Chek; Roche Diagnostics India, Mumbai, India), which were used for calibration at the time of iPro2 data upload. On the day after the 4 day regimen, following an overnight fast, blood was collected for glucose, insulin and lipid measurements. For each participant, blood was procured at the exact same time during each regimen (between 08:30 and 09:30 hours).

### Assessment of physical activity and estimated energy expenditure

Physical activity was measured 24 h/day using an activPAL3 monitor (PAL Technologies, Glasgow, Scotland). The activPAL was waterproofed with a small sleeve to cover the monitor, wrapped in one piece of medical-grade adhesive dressing (Tegaderm; 3M, Saint Paul, MN, USA) and then attached to the anterior thigh using Tegaderm (3M) on the first morning of each activity regimen. As the activPAL data from day 1 did not cover the entire day, only activPAL data from the last 72 h of each regimen were analysed. This accelerometer accurately discriminates between time spent inactive (sitting or lying), standing and walking [19], giving step number and cadence [20]. Diary data for self-reported physical activity were compared with the activPAL data after the first and third days of each activity regimen to formulate tailor-made instructions on how to alter daily activities in order to guarantee optimal compliance with each activity regimen. Time spent sleeping was determined based on the diary data.

Daily energy expenditure was estimated using the 24 h activPAL data, which yielded this information as metabolic equivalents (METs). The measurement error of the activPAL in estimating this energy expenditure has been reported to be low for sedentary and light-intensity activities but high for moderate- and vigorous-intensity activities [21]. Thus, the *Compendium of Physical Activities* [22] was used to determine the energy expenditure of cycling by generating a linear regression line from studies in the compendium that provided MET values over a range of workloads representative of the population in our study. Consequently, the energy expenditure of cycling was determined using the following equation: MET = 0.0545 × watt + 1.4561; *R* ^2^ = 0.946, using each participant’s workload in watts to determine individual energy expenditure. The Exercise and Sit Less regimens were designed to have comparable energy expenditure. To achieve this, during screening, every individual performed a 1 day try-out of the Sit Less regimen in free-living conditions, a maximal workload capacity (W_max_) test (a progressive cycle test until exhaustion while cardiac function was monitored) and an additional 1 h supervised cycle test, each on separate days. Estimated energy expenditure, as derived from the activPAL data during the Sit Less try-out day, was used as the input for computing the duration (∼1 h) and workload (50–60% W_max_) for cycling during the Exercise regimen. Normal daily activities were measured 13 days prior to the start of the study (‘day −13’) and participants were asked not to cycle on this day.

### Data processing and statistical analysis

All data were double-entered. The continuous glucose monitoring data were analysed using an iPro2 (Medtronic). The last 24 h of each regimen, starting with the first fingerprick before breakfast, were chosen for analysis of the 24 h glucose levels since meals were identical between interventions during this period. Mean 24 h glucose concentration was defined as the average glucose of 288 measurements equally spaced in time. The incremental AUC (iAUC) for glucose was calculated using the trapezoid rule [23]. The iAUC provides a summary measure of the increase above fasting glucose level during the subsequent 24 h observation period. A secondary outcome was total AUC (all values above zero). Hyperglycaemia was defined as a glucose level of ≥10.0 mmol/l, whilst hypoglycaemia was defined as a glucose concentration ≤3.9 mmol/l plus clinical symptoms.

To examine whether bouts of cycling or walking resulted in a rapid decrease in glucose levels, the average glucose decrease during each 30 min time frame was calculated using continuous glucose monitoring, resulting in 288 values over 24 h.

The HOMA2 computational method (www.dtu.ox.ac.uk, accessed 1 December 2015) [24] was used to estimate insulin resistance (HOMA2-IR) using fasting plasma glucose and insulin values measured on the day after completion of the 4 day regimen.

All statistical calculations were performed using IBM SPSS Statistics for Windows (Version 21; Armonk NY, USA). The differences in outcome between regimens were analysed using linear mixed model analyses with activity regimen and period (order of activity regimen) as fixed factors, and an unstructured covariance structure for the three repeated measurements for each person. Natural log transformation was performed if the outcome was not normally distributed. Likelihood-based methods were used without imputing missing outcome values. Numerical variables are presented as mean ± SD, or as median (first quartile, third quartile) for baseline characteristics (measured during screening) and estimated mean (SEM) for the other values. A *p* value of ≤0.05 was considered statistically significant. When regimens were compared pairwise, *p* values ≤0.017 (=0.05/3, Bonferroni correction) were considered statistically significant to account for multiple testing (three pairwise comparisons).

## Results

After screening, 20 participants (14 men, six women) were included in the study (see electronic supplementary material [ESM] Fig. 1 for recruitment flow chart). Before completing the protocol, one participant withdrew because of osteoarthritis-related pain during walking. The remaining 19 individuals had a mean age of 63 years and a mean BMI of 30.5 kg/m^2^ (Table 1). The median duration of type 2 diabetes was 6 years and participants had a mean HbA_1c_ of 6.7% (49.5 mmol/mol) and mean fasting plasma glucose of 7.88 mmol/l during screening (Table 1). Fourteen participants were using oral glucose-lowering drugs (metformin, *n* = 14; sulfonylurea, *n* = 7; sitagliptin, *n* = 2), and 13 were using lipid-lowering drugs (statins, *n* = 12; ezetimibe, *n* = 2).

**Table 1:** Participant characteristics

Variables	Mean (SD)
*N*	19
Men (*n*)	13
Age (years)	63 (9)
Duration of type 2 diabetes (years)^a^	6 (4, 10)
Glucose-lowering drugs (*n*)	14
Lipid-lowering drugs (*n*)	13
Height (m)	1.70 (0.07)
Weight (kg)	88.8 (12.0)
BMI (kg/m^2^)	30.5 (3.3)
Waist circumference (cm)	105 (8)
W_max_ (W)	152 (43)
Systolic BP (mmHg)	143 (12)
Diastolic BP (mmHg)	82 (8)
Glucose (mmol/l)	7.88 (1.51)
Triacylglycerol (mmol/l)	1.51 (0.55)
HbA_1c_ (%)	6.7 (0.8)
HbA_1c_ (mmol/mol)	49.5 (8.8)

Data are expressed as mean (SD) or ^a^median (first quartile, third quartile)

### Continuous glucose monitoring

**Fig. 2:**
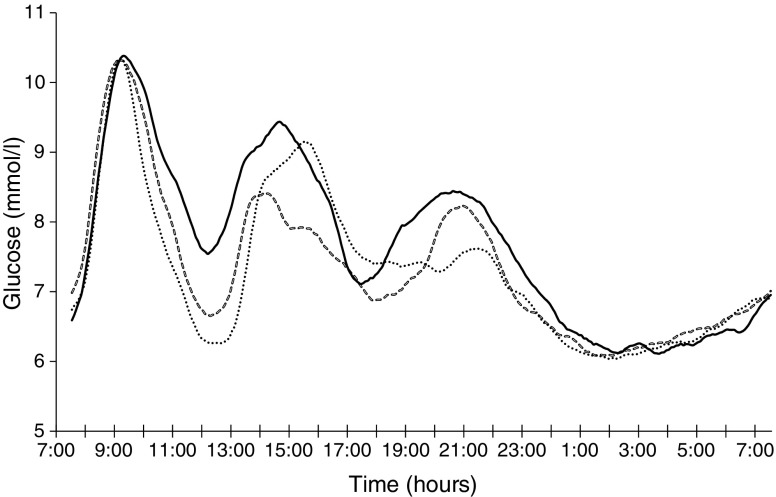
Mean 24 h glucose profiles during the last day of each activity regimen (*n* = 19 individuals). Solid line, Sitting regimen; dashed line, Sit Less regimen; dotted line, Exercise regimen

**Fig. 3:**
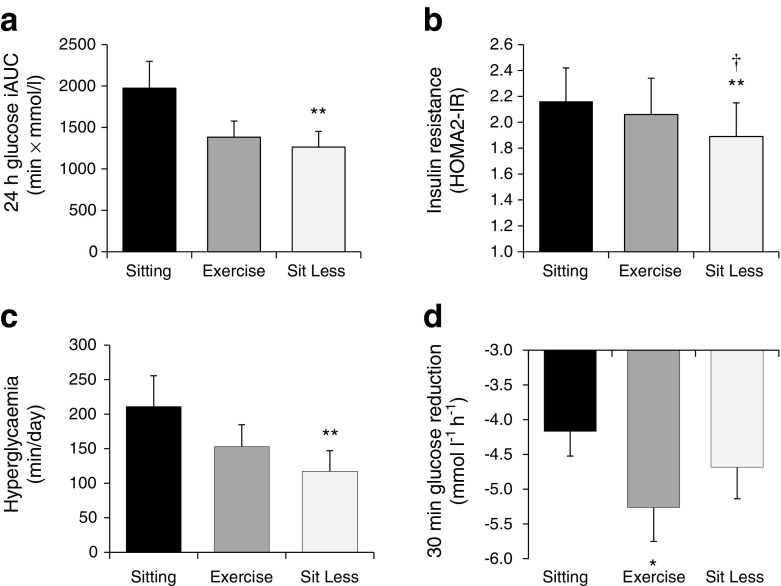
(**a**) Twenty-four hour glucose iAUC during the last day of each activity regimen, (**b**) insulin resistance expressed as HOMA2-IR on the morning after each activity regimen, (**c**) duration of hyperglycaemia, and (**d**) maximal reduction in glucose level at 30 min during the last day of each activity regimen. Data are estimated means ± SEM (*n* = 19 individuals). * *p* ≤ 0.05, **p < 0.01 vs Sitting regimen; †, *p* ≤ 0.05 vs Exercise regimen

In the Sit Less regimen, substitution of time spent sitting by ambulatory time significantly reduced 24 h glucose levels compared with Sitting (mean [SEM] Sit Less vs Sitting: 7.35 [0.19] vs 7.69 [0.23] mmol/l; *p* = 0.014; Figs 2 and 3a). In contrast, 24 h glucose levels were not significantly different between Sit Less and the more classical Exercise regimen, involving structured exercise (Exercise: 7.29 [0.24] mmol/l; *p* = 0.741). Similar results were obtained for total AUC (Table 2). Sit Less profoundly reduced glucose levels (by approximately 36%) compared with the Sitting regimen; i.e. the iAUC for 24 h glucose levels reduced significantly from 1974 (324) min × mmol/l in the Sitting regimen, to 1263 (189) min × mmol/l in the Sit Less regimen (*p* = 0.002; Figs 2 and 3a). Glucose excursion (iAUC) for 24 h glucose was similar between Sit Less and Exercise (*p* = 0.499). Although structured exercise also reduced the 24 h glucose excursion (iAUC 1383 [194] min × mmol/l; *p* = 0.069) compared with Sitting, this effect was not significant and, hence, less pronounced than in the Sit Less regimen.

**Table 2:** Effect of activity regimens on blood parameters

Variables	SIT	EXE	SL	*p* value^b^	*p* EXE-SIT^c^	*p* SL-SIT^c^	*p* SL-EXE^c^
Glucose (mmol/l)	7.35 (0.23)	7.60 (0.26)	7.29 (0.25)	0.121	0.046	0.616	0.086
Insulin (pmol/l)	108 (13)	102 (14)	95 (14)	0.005	0.117	0.001	0.023
C-peptide^a^ (nmol/l)	0.06 (0.08)	0.01 (0.08)	−0.03 (0.09)	0.051	0.165	0.016	0.269
Triacylglycerol (mmol/l)	1.93 (0.17)	1.55 (0.14)	1.46 (0.12)	0.002	0.003	<0.001	0.200
Total cholesterol (mmol/l)	5.24 (0.30)	5.10 (0.25)	5.06 (0.23)	0.253	0.439	0.194	0.611
Non-HDL-C (mmol/l)	3.89 (0.32)	3.66 (0.30)	3.65 (0.27)	0.071	0.212	0.047	0.890
LDL-C (mmol/l)	3.02 (0.29)	2.96 (0.27)	2.99 (0.24)	0.946	0.751	0.813	0.770
HDL-C (mmol/l)	1.35 (0.11)	1.44 (0.11)	1.41 (0.10)	0.198	0.082	0.213	0.340
Apo B (g/l)	0.91 (0.07)	0.87 (0.06)	0.88 (0.06)	0.514	0.365	0.314	0.897
NEFA (mmol/l)	0.48 (0.05)	0.65 (0.06)	0.59 (0.04)	0.028	0.017	0.012	0.201
Free glycerol (mmol/l)	0.11 (0.01)	0.12 (0.01)	0.11 (0.01)	0.288	0.836	0.557	0.127
24 h glucose AUC (min × mmol/l)	11,071 (334)	10,503 (342)	10,589 (268)	0.035	0.064	0.013	0.742

Data (*n* = 19 individuals) are presented as estimated means (SEM)

All values are fasting blood parameters assessed on the morning following completion of each activity regimen, except for 24 h glucose AUC which was assessed during day 4 of each activity regimen

^**a**^C-peptide analyses were carried out following natural log transformation

*p* values were generated using linear mixed model analyses for ^b^overall difference and ^c^pairwise comparisons between activity regimens

EXE, Exercise; HDL-C, HDL-cholesterol; LDL-C, LDL-cholesterol; SIT, Sitting; SL, Sit Less

While chronic elevated glucose levels are associated with adverse health effects, the frequency and duration of hyperglycaemia (min/day that blood glucose is ≥10 mmol/l) may be of even more relevance in a clinical setting [25]. The duration of hyperglycaemia over a 24 h time span was almost halved (Fig. 3c), from 211 (44) min/day in the Sitting regimen to 118 (32) min/day in the Sit Less regimen (*p* = 0.002). Again, intermediate results were observed for the Exercise regimen, with the duration of hyperglycaemia averaging 152 (30) min/day. Although no hypoglycaemic periods were observed during any of the regimens, the maximal fall in glucose within 30 min differed between experimental regimens (Fig. 3d). As anticipated, the maximal drop in glucose over 30 min during structured exercise (−5.28 [0.39] mmol l^−1^ h^−1^) was larger than during the Sitting regimen (−4.15 [0.31] mmol l^−1^ h^−1^; *p* = 0.016). Importantly, the maximal blood glucose reduction observed in the Sit Less regimen (−4.69 [0.39] mmol l^−1^ h^−1^) was not significantly different from that observed after Sitting (*p* = 0.119), indicating that the Sit Less regimen is unlikely to increase the risk of experienced hypoglycaemia.

### Insulin resistance (HOMA2-IR) and plasma lipids

Fasting insulin levels in the Exercise regimen were similar to those in the Sitting regimen (102 [14] vs 108 [13] pmol/l, respectively; *p* = 0.117). In contrast, compared with Sitting, Sit Less significantly (*p* = 0.001) reduced fasting insulin levels to 95 [14] pmol/l). Since no significant differences were observed in mean glucose values after Sit Less as compared with Exercise (Table 2), this finding suggests that Sit Less, but not Exercise, improved insulin sensitivity. Likewise, Sit Less resulted in significantly lower HOMA2-IR values vs Sitting (1.89 [0.26] vs 2.16 [0.26]; *p* = 0.001) and vs Exercise (2.06 [0.28]; *p* = 0.015), whereas Exercise failed to improve this measure of insulin sensitivity (Fig. 3b) compared with the Sitting regime (*p* = 0.177).

Both Sit Less (1.46 [0.12] mmol/l and Exercise (1.55 [0.14] mmol/l) resulted in significantly lower fasting triacylglycerol levels than were observed after Sitting (1.93 [0.17] mmol/l; both *p* < 0.01). We also observed that after both Sit Less and Exercise, fasting plasma NEFA levels were significantly higher (Sit Less 0.59 [0.04] mmol/l, *p* = 0.012; Exercise 0.65 [0.06] mmol/l, *p* = 0.017) than after Sitting (0.48 [0.05] mmol/l). Cholesterol-related variables and free glycerol were not significantly different between any of the regimens (Table 2).

### Physical activity and diet

Before the start of the study, the average time spent walking, standing and sitting/sleeping in free-living conditions (*n* = 16 individuals; activPAL data were not available for three participants) was 1.4 (0.2), 3.6 (0.5) and 18.9 (0.6) h/day, respectively (data not shown). During each regimen, we successfully manipulated the time spent sitting, walking, standing and cycling so that they were close to the times stipulated for each activity/regimen in the protocol (Table 3). During Sit Less, the times spent walking (∼3 h/day) and standing (∼4 h/day) were significantly longer than in the other regimens, whereas Sitting and Exercise were not significantly different with respect to the time spent walking (∼1 h/day) and standing (∼1.6 h/day). During the Exercise regimen, on average 65 (3) min of sitting per day were substituted by cycling at an intensity of 81 (4) W, corresponding to a mean of 5.9 (0.2) MET. Walking cadence was 93 (2) steps/min during Sit Less and 83 (2) and 84 (2) during Sitting and Exercise, respectively (Table 3). By design, estimated energy expenditure was lower in the Sitting regimen (32.0 [0.1] MET × h/day) than in any of the other regimens (36.9 [0.2] and 37.4 [0.2] MET × h/day for Exercise and Sit Less, respectively; Table 3). There was, however, an estimated 0.5 MET × h/day difference between Exercise and Sit Less (*p* = 0.008; Table 3). Energy intake did not differ significantly between the regimens (*p* = 0.599 during activity regimens and *p* = 0.953 during the last day of each activity regimen) and neither did the percentage macronutrients consumed (Table 3 and ESM Table 1).

**Table 3:** Physical activity and diet analysis during activity regimens

Variables	SIT	EXE	SL	*p* value^a^	*p* EXE-SIT^b^	*p* SL-SIT^b^	*p* SL-EXE^b^
Estimated EE (MET × h/day)	32.0 (0.1)	36.9 (0.2)	37.4 (0.2)	<0.001	<0.001	<0.001	0.008
Energy intake (kJ)	7525 (288)	7632 (263)	7591 (283)	0.599	0.318	0.412	0.516
Carbohydrates (%)	47.1 (1.0)	47.6 (0.7)	47.3 (0.6)	0.272	0.338	0.806	0.409
Protein (%)	18.5 (0.6)	18.6 (0.5)	18.6 (0.5)	0.932	0.727	0.718	0.853
Fat (%)	34.5 (1.0)	34.0 (0.9)	34.0 (0.8)	0.608	0.328	0.470	0.798
Sitting (h/day)	13.7 (0.3)	12.5 (0.2)	8.9 (0.3)	<0.001	<0.001	<0.001	<0.001
Walking (h/day)	0.9 (0.1)	1.0 (0.1)	3.1 (0.1)	<0.001	0.050	<0.001	<0.001
Standing (h/day)	1.6 (0.1)	1.6 (0.1)	4.1 (0.2)	<0.001	0.996	<0.001	<0.001
Cycling (h/day)	–	1.1 (0.1)	–	–	–	–	–
Sleeping (h/day)	7.9 (0.2)	7.9 (0.2)	7.9 (0.2)	0.957	0.810	0.919	0.863
Steps/day (*n*)	4415 (298)	4823 (241)	17,502 (620)	<0.001	0.035	<0.001	<0.001
Cadence (steps/min)	83 (2)	84 (2)	93 (2)	<0.001	0.281	<0.001	<0.001

Data (*n* = 19 individuals) are presented as estimated means (SEM)

Diet during the activity regimens was assessed via diary data (from all 4 days of each regimen); activities were assessed during the last 3 days of each activity regimen using the activPAL accelerometer

*p* values were generated using linear mixed model analyses for ^a^overall difference and ^b^pairwise comparisons between activity regimens

EE, Energy expenditure; EXE, Exercise; SIT, Sitting; SL, Sit Less

## Discussion

Structured exercise is a recognised cornerstone of type 2 diabetes treatment and prevention. However, sustained compliance with exercise programmes, especially by individuals with type 2 diabetes, is at best mediocre [26, 27]. Observational studies have revealed associations between the time spent sitting and markers of metabolic disturbance [8–10]. Therefore, reducing the sitting time may improve glycaemic control and insulin sensitivity in type 2 diabetes. Strategies to reduce sitting time are generally considered to be less demanding than structured exercise programmes and hence are more likely to have long-term compliance [28]. In this study, we observed that the Sit Less regimen improved insulin sensitivity, mean 24 h glucose levels, 24 h glucose excursions, duration of hyperglycaemia (blood glucose ≥10 mmol/l) and fasting triacylglycerol levels.

We used a proof-of-concept study to determine the relative efficacy of reducing sitting time or increasing structured exercise. As a result, the number of steps during Sit Less (around 17,500 steps/day) was well above what is generally observed in patients with type 2 diabetes (around 6500–8000 steps/day) [7, 29] and, therefore, the duration/intensity of exercise was also high (65 min/day cycling at 5.9 MET) and probably not sustainable on a long-term basis. The beneficial effects of Sit Less were observed in participants with type 2 diabetes, the majority of whom were men using oral glucose-lowering medication (the use of which was continued throughout the study duration). Subanalyses showed similar improvements in 24 h glucose iAUC during Sit Less in participants who were not taking glucose-lowering medication (*n* = 5), as well as for women only (*n* = 6).

The outcome of the present study fits the emerging picture that breaking up sedentary behaviour by light-intensity activities may help to improve glucose homeostasis in groups, ranging from young lean normoglycaemic individuals [16] to overweight/obese normoglycaemic [13] and dysglycaemic [14] participants. Very recently, Dempsey et al showed that breaking up sitting time with brief bouts of light-intensity walking or resistance exercise attenuates postprandial glucose and insulin responses in type 2 diabetes [12]. In the current study, the general effect of the Sit Less regimen on glucose homeostasis tended to be a little more potent than the effect of structured exercise. One of the effects of acute exercise on elevated blood glucose in diabetes is a post-exercise reduction in glucose levels; this can sometimes result in hypoglycaemia and can render some diabetic patients reluctant to perform structured exercise. In our study, the Sit Less regimen did not result in glucose level reductions at 30 min compared with the Sitting regimen, whereas 30 min glucose levels fell by more than 5 mmol l^−1^ h^−1^ during the Exercise regimen. These data suggest that more stable glucose levels can be achieved with light-intensity activity rather than with exercise.

For most variables, other than 24 h glucose, the structured exercise regimen (Exercise) had beneficial effects. The effect size of Exercise, however, was typically intermediate compared with the effects of Sit Less and was only greater for the increases observed in fasting plasma NEFA levels vs Sitting. These results are in line with a recent study by Henson et al showing that breaking up sitting time with walking or standing increased NEFA levels in overweight/obese postmenopausal women [14]. Although the mechanism underlying the increase in NEFA concentration after Sit Less and Exercise is incompletely understood, it probably reflects a spill-over of fatty acids from increased adipose tissue lipolysis used to fuel contractile activity. Upon cessation of contractile activity, the amount of fatty acids taken up by the muscle for oxidation decreases more promptly than the catecholamine-induced elevation of adipose tissue lipolysis, resulting in elevated plasma NEFA levels [30]. In contrast to our previous study in healthy volunteers [16], we failed to observe significant effects on cholesterol levels in individuals with type 2 diabetes. The most likely explanation for this apparent discrepancy is the fact that participants stopped lipid-lowering medication 2 weeks prior to the study, a period that may have been too short.

Both Sit Less and Exercise resulted in lower fasting plasma triacylglycerol levels, which may be the result of increased triacylglycerol clearance due to enhanced lipoprotein lipase activity [31]. These observed lower triacylglycerol levels after physical activity are in line with previous [16, 31] but not all [15] studies. In the latter study, however, triacylglycerol levels were probably measured too soon after exercise, as lipoprotein lipase activity typically peaks ≥8 h post exercise.

In line with our previous study in healthy individuals [16], we observed that insulin sensitivity was more greatly improved after the Sit Less regimen than after the Exercise regimen. This finding may seem surprising given the high dose of exercise (65 min/day cycling at an intensity of 5.9 MET). It is possible that HOMA-IR was not accurate enough to detect relatively small changes in insulin sensitivity after Exercise. However, it should be noted that, during the Exercise regimen, participants spent most of the day sitting. We observed similar findings in healthy individuals [16] and we therefore suggest that one bout of exercise probably cannot fully compensate for the negative effects of sitting for the rest of the day. Thus, the duration of non-sitting activities may be more important than the intensity of these activities. Since the participants were instructed to break up sitting time every 30 min, this may have contributed to the beneficial effects observed during Sit Less in comparison with Exercise. However, the activPAL programme did not provide information on the duration of sitting bouts; therefore, this behavioural outcome could not be well assessed. Energy expenditure was expressed in METs by the activPAL; for the sake of comparison, we also converted the external workload on the stationary bike to MET values. Although we appreciate that the conversion of measured power output to predicted MET values is a proxy for the actual energy expenditure, we believe that this method is valid when it comes to comparing the energy expenditure in Sit Less with Exercise.

In conclusion, this study suggests that breaking up sitting time by promoting time spent standing and light-intensity walking is a potent way to beneficially affect insulin resistance and other clinically relevant markers of glucose metabolism, and plasma triacylglycerols in individuals with type 2 diabetes who are taking oral glucose-lowering medication. Although an approximate energy-matched intervention with structured exercise also displayed most of the beneficial effects of breaking up sitting time, the present study provides indications favouring the implementation of interventions targeting the breaking-up of sitting time over interventions involving structured exercise. The effect of breaking up sitting time on insulin resistance was more pronounced than that of structured exercise. Additionally, the more abrupt and prominent reduction in blood glucose in the structured exercise intervention is proposed to increase the risk of hypoglycaemia [32]. Since the volume of activities in this proof-of-concept study was high, future long-term studies are needed to determine the volume of light-intensity activities that is feasible in daily life.

## Electronic supplementary material

Below is the link to the electronic supplementary material.

ESM (PDF 191 kb)

## Abbreviations

iAUC: Incremental AUC
MET: Metabolic equivalent
W_max_: Maximal workload capacity
