# Supplementary material for: Breaking sitting with light activities vs structured exercise: a randomised crossover study demonstrating benefits for glycaemic control and insulin sensitivity in type 2 diabetes
Source: Diabetologia. 2016 Nov 30;60(3):490–8. doi: 10.1007/s00125-016-4161-7 (PMC6518091; doi:10.1007/s00125-016-4161-7)
Supplement: Supplementary file 1 — (PDF 191 kb) [file 125_2016_4161_MOESM1_ESM.pdf]

**ESM Table 1** Physical activity and diet analysis during last day of each activity regimen

| Variables | SIT | EXE | SL | *p* value[a] | *p* EXE-SIT[b] | *p* SL-SIT[b] | *p* SL-EXE[b] |
|---|---|---|---|---|---|---|---|
| **Estimated EE (MET × h/day)** | 32.0 (0.1) | 36.9 (0.2) | 37.5 (0.3) | <0.001 | <0.001 | <0.001 | 0.056 |
| **Energy intake (kJ)** | 9340 (299) | 9367 (284) | 9351 (293) | 0.953 | 0.779 | 0.891 | 0.792 |
| **Carbohydrates (%)** | 52.1 (0.7) | 52.4 (0.6) | 52.5 (0.6) | 0.332 | 0.400 | 0.253 | 0.315 |
| **Protein (%)** | 17.2 (0.4) | 17.2 (0.4) | 17.0 (0.4) | 0.228 | 0.822 | 0.498 | 0.116 |
| **Fat (%)** | 30.5 (0.9) | 30.3 (0.8) | 30.2 (0.8) | 0.438 | 0.560 | 0.253 | 0.590 |
| **Sitting (h/day)** | 13.7 (0.4) | 13.0 (0.3) | 9.0 (0.4) | <0.001 | 0.162 | <0.001 | <0.001 |
| **Walking (h/day)** | 0.9 (0.1) | 0.9 (0.1) | 3.2 (0.1) | <0.001 | 0.692 | <0.001 | <0.001 |
| **Standing (h/day)** | 1.5 (0.1) | 1.3 (0.1) | 3.9 (0.2) | <0.001 | 0.181 | <0.001 | <0.001 |
| **Cycling (h/day)** | - | 1.1 (0.1) | - | - | - | - | - |
| **Sleeping (h/day)** | 7.9 (0.3) | 7.7 (0.2) | 8.0 (0.3) | 0.372 | 0.444 | 0.886 | 0.169 |
| **Steps/day (n)** | 4476 (321) | 4595 (244) | 17,993 (654) | <0.001 | 0.708 | <0.001 | <0.001 |
| **Cadence (steps/min)** | 84 (2) | 84 (2) | 95 (2) | <0.001 | 0.826 | <0.001 | <0.001 |

Data (*n*=19 individuals) are presented as estimated means (SEM)

Diet and activities (during last day of each activity regimen) were assessed via diary data and activPAL accelerometry respectively

*P* values were generated using linear mixed model analyses for [a]overall difference and [b]pairwise comparisons between activity regimens

EE, Energy expenditure; EXE, Exercise; SIT, Sitting; SL, Sit Less

## ESM Fig. 1: Trial CONSORT diagram

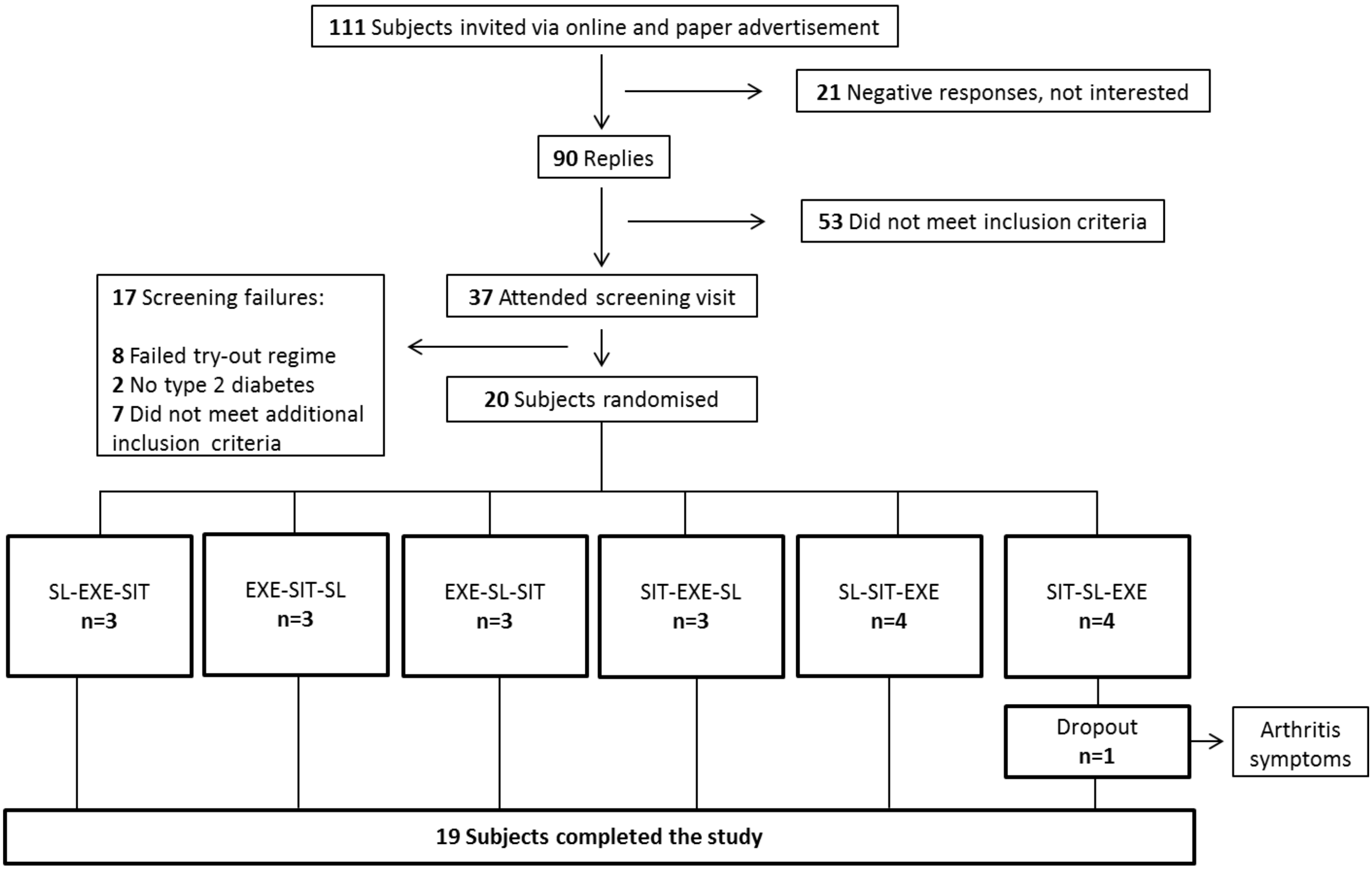


EXE, Exercise; SIT, Sitting; SL, Sit Less
